# Dr. John Clark LeGrand

**Published:** 1906-05

**Authors:** 


Dr. John Clark Le Grand, of Birmingham, Ala., died at his home in that city, March 21, 1906, aged 52 years. He had been in impaired health for more than a year with leukemia, but his death was, at last, somewhat sudden. Dr. Le Grand was the founder, editor and publisher of the Alabama Medical Journal and was professor of obstetrics in the Birmingham Medical College. He occupied a position of prominence among the medical profession of Alabama, and had been president of the State Medical Association. He was a dignified, amiable man, who will be greatly missed by his colleagues as well as by the community in general.

